# Whole-genome sequencing of a *Plasmodium vivax* isolate from the China-Myanmar border area

**DOI:** 10.1590/0074-02760150216

**Published:** 2015-09

**Authors:** Hai-Mo Shen, Shen-Bo Chen, Yue Wang, Jun-Hu Chen

**Affiliations:** 1 National Institute of Parasitic Diseases, Chinese Center for Disease Control and Prevention, Key Laboratory of Parasite and Vector Biology, Chinese Ministry of Health, WHO Collaborating Center for Malaria, Schistosomiasis and Filariasis, Shanghai, People’s Republic of China; 2 Institute of Parasitic Diseases, Zhejiang Academy of Medical Sciences, Hangzhou, People’s Republic of China

**Keywords:** Plasmodium vivax, genome, vir

## Abstract

Currently, there is a trend of an increasing number of* Plasmodium vivax*malaria cases in China that are imported across its Southeast Asia border, especially in the China-Myanmar border area (CMB). To date, little is known about the genetic diversity of* P. vivax *in this region. In this paper, we report the first genome sequencing of a* P. vivax*isolate (CMB-1) from a vivax malaria patient in CMB. The sequencing data were aligned onto 96.43% of the *P. vivax *Salvador I reference strain (Sal I) genome with 7.84-fold coverage as well as onto 98.32% of 14 Sal I chromosomes. Using the* de novo *assembly approach, we generated 8,541 scaffolds and assembled a total of 27.1 Mb of sequence into CMB-1 scaffolds. Furthermore, we identified all 295 known* vir*genes, which is the largest subtelomeric multigene family in malaria parasites. These results provide an important foundation for further research on*P. vivax *population genetics.

As the most common human malaria species with the widest geographic distribution,*Plasmodium vivax* is mostly found outside of Africa and is especially prevalent in Southeast Asia and America (Price et al. 2007). The *P. vivax* parasite is now considered the cause of severe malaria syndromes that have been blamed on *P. falciparum* (Price et al. 2009). It was estimated that half of the world’s population is at risk of *P. vivax* malaria (Guerra et al. 2010).

In China, *P. vivax* was the major species for a relatively long time. The Yunnan province remains the highest transmission area in China, particularly in the southern border areas adjacent to Myanmar, a highly endemic area for *P. vivax* malaria in the Greater Mekong Subregion countries (Zhou et al. 2014). Due to the increasing numbers of Chinese working abroad, the number of imported *P. *vivax cases has exhibited an increasing trend in recent years (Feng et al. 2015). The imported *P. vivax* malaria may lead to high malaria risk in malaria-free localities where the *Anopheles sinensis*mosquito is prevalent, particularly in central China, such as in Anhui and Henan provinces (Gao et al. 2004). In 2012, 1,143*P. vivax* malaria cases were reported in China, accounting for 41.9% of the total malaria cases.

Hundreds of *P. falciparum* isolates have been sequenced or genotyped (Winzeler 2008), but less information on*P. vivax* isolates has been reported. The first complete genome of*P. vivax* was published in 2008 (Carlton et al. 2008), revealing that *P. vivax* resembles other *Plasmodium* spp in gene content and metabolic traits, but it possesses novel gene families and potential alternative invasion pathways not previously been recognised. Although they were started more recently, there are several *P. vivax* isolate genome sequencing projects underway and more sequence data have been made available (Neafsey et al. 2012). Previously, 23 isolates genome data were accessible in the National Center for Biotechnology Information (NCBI) database. However, no isolates from China or China’s borders with Southeast Asian countries have previously been included.

In this paper, we report the first *P. vivax* genome sequence of a clinical isolate obtained in the China-Myanmar border area (CMB-1) as well as in the China-Southeast Asia border areas. The genomic DNA of the *P. vivax*CMB-1 for sequencing was extracted from a whole blood sample from a patient with microscopically positive for *P. vivax* and polymerase chain reaction confirmed sole infection with *P. viva*x. The Ethical Committee of the National Institute of Parasitic Diseases, China Center for Disease Control and Prevention approved the study (NIPD 2013-010). The study protocol, potential risks and potential benefits were explained to the patient and informed consent was verbally obtained. The genomic DNA was used to construct the Illumina sequencing library with insert sizes of 360 bp. The library was sequenced on a HiSeq 2000 sequencer. After filtering the sequences as for the *Homo sapiens* genome, the reads were*de novo* assembled using an A5 assembly pipeline (Coil et al. 2015). The Illumina sequencing reads have been submitted to the NCBI Short Read Archive (accession SRS941624).

The whole genome sequencing generated 31,471,932 paired-end reads with an average read length of 125 bp. Low-quality bases and adapters were trimmed using Trimmomatic v.0.30 (Bolger et al. 2014). The sequence reads were aligned to the *P. vivax* Salvador I reference strain (Sal I) genome using BWA-0.7.1 (Li & Durbin 2009). In total, 5.86% of 26 million quality-evaluated reads were aligned onto 96.43% of the Sal I genome with 7.84-fold coverage as well as onto 98.32% of the 14 chromosomes of the Sal I strain covering 95.96-99.05% for each chromosome.

The *de novo* assembly yielded a database with 8,541 scaffolds (10,639 contigs) and an average guanine-cytosine content of 39.1%. A total sequence coverage of 10.26-fold produced this assembly with N50 scaffold lengths of 5.9 kb. A total of 27.1 Mb of sequence was assembled in the CMB-1 scaffolds (Table).

**Figure:**
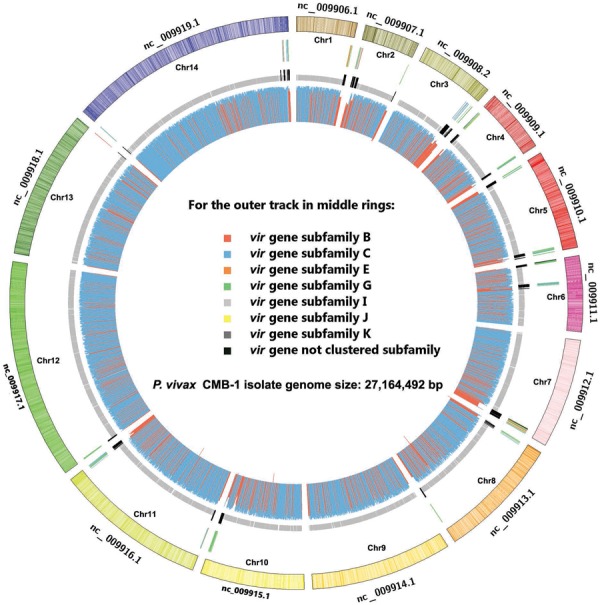
Genomic map of *Plasmodium vivax* China-Myanmar border area (CMB)-1 isolate. The outermost circle shows the whole genome of*P. vivax* Salvador I reference strain. The next ring represents the location of 295 *vir* genes, each subfamily marked in different colour. The third ring shows the coding sequences zone of CMB-1 isolate scaffolds and v*ir* gene orthologs are indicated by black. The inner circle shows the genomic map of *P. vivax* CMB-1 isolate and the histogram represents the degree of similarity (blue: identity > 99%). The figure was drawn using Circos (Krzywinski et al. 2009) and alignment was performed using MUMmer 3.0 (Kurtz et al. 2004).

**TABLE:** *De novo* assembly statistics of the *Plasmodium vivax* China-Myanmar border area-1 genome

Attribute	
Raw reads	15,105,614 paired
Unmapped reads to *Homo sapiens*	1,268,041 paired
After quality control	1,267,552 paired
Contigs (n)	10,639
Scaffolds (n)	8,541
Longest scaffold (bp)	125,157
N50 (bp)	5,936
Genome size (bp)	27,164,492
Coverage	10.26

An overall comparative genomic analysis was conducted using the complete genome of the*P. vivax* reference strain Sal I, as shown in Figure. It is widely known that the *vir* super-family is variably expressed and encodes proteins that are exported to the host cell surface to evade the host adaptive immune response (Fernandez-Becerra et al. 2009). As the largest subtelomeric multigene family of malaria parasites, the *vir*super-family consists of seven different subfamilies. In the CMB-1 *de novo* assembled sequences, we identified all published 295*vir* genes (Lopez et al. 2013) based on their sequence similarity in BLASTX.

The findings in this paper provide whole genomic information on the current epidemiological scenario of vivax malaria in the CMB, where the number of *P. vivax* cases imported from Southeast Asia is increasing and accompanied by growing concern. The results of this work contribute to a better understanding of*P. vivax* evolution and provide an informative basis for further study of the population genomics of this parasite.
